# EUS-guided jejuno-enterostomy in a patient with total gastrectomy with Roux-en-Y esophagojejunostomy to facilitate cholangioscopy with electrohydraulic lithotripsy

**DOI:** 10.1016/j.vgie.2022.08.024

**Published:** 2022-10-17

**Authors:** Yervant Ichkhanian, Hamna Fahad, Mouhanna Abu Ghanimeh, Tobias Zuchelli

**Affiliations:** 1 Department of Medicine, Henry Ford Hospital, Detroit, Michigan; 2 Division of Gastroenterology and Hepatology, Henry Ford Hospital, Detroit, Michigan; 3 Division of Gastroenterology and Hepatology, Stanford Health, Sioux Falls, South Dakota; 4 Division of Gastroenterology and Hepatology, Henry Ford Hospital, Detroit, Michigan

**Keywords:** EHL, electrohydraulic lithotripsy, LAMS, lumen-apposing metal stent, LFT, liver function test

## Abstract

Video 1 EUS-guided jejuno-jejunostomy in a 67-year-old male patient with total gastrectomy with Roux-en-Y esophagojejunostomy to facilitate cholangioscopy with electrohydraulic lithotripsy.

EUS-guided jejuno-jejunostomy in a 67-year-old male patient with total gastrectomy with Roux-en-Y esophagojejunostomy to facilitate cholangioscopy with electrohydraulic lithotripsy.

## Introduction

**Figure 1:**
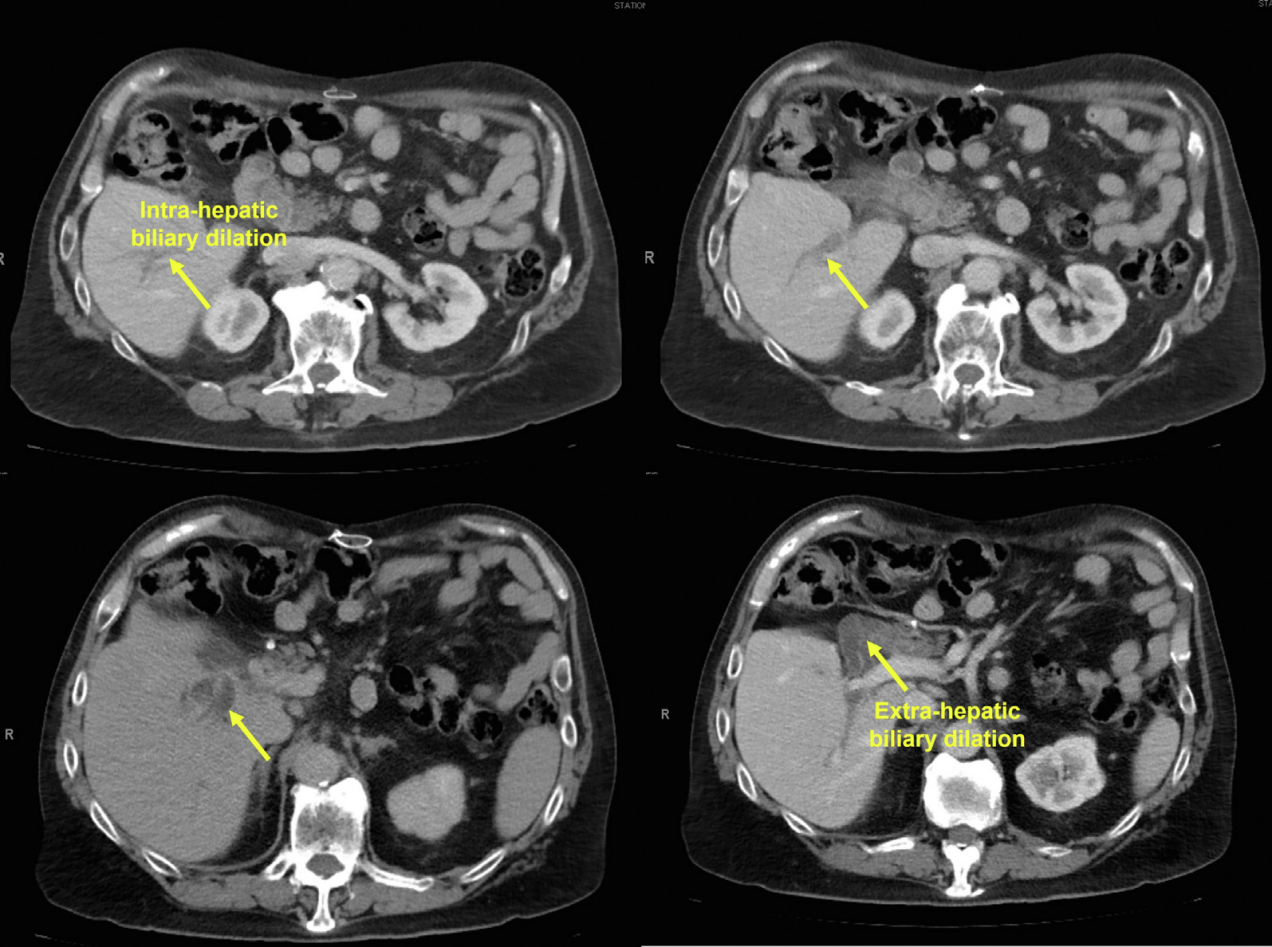
Transverse plane liver CT scan indicating dilation of the intrahepatic and extrahepatic bile ducts.

**Figure 2:**
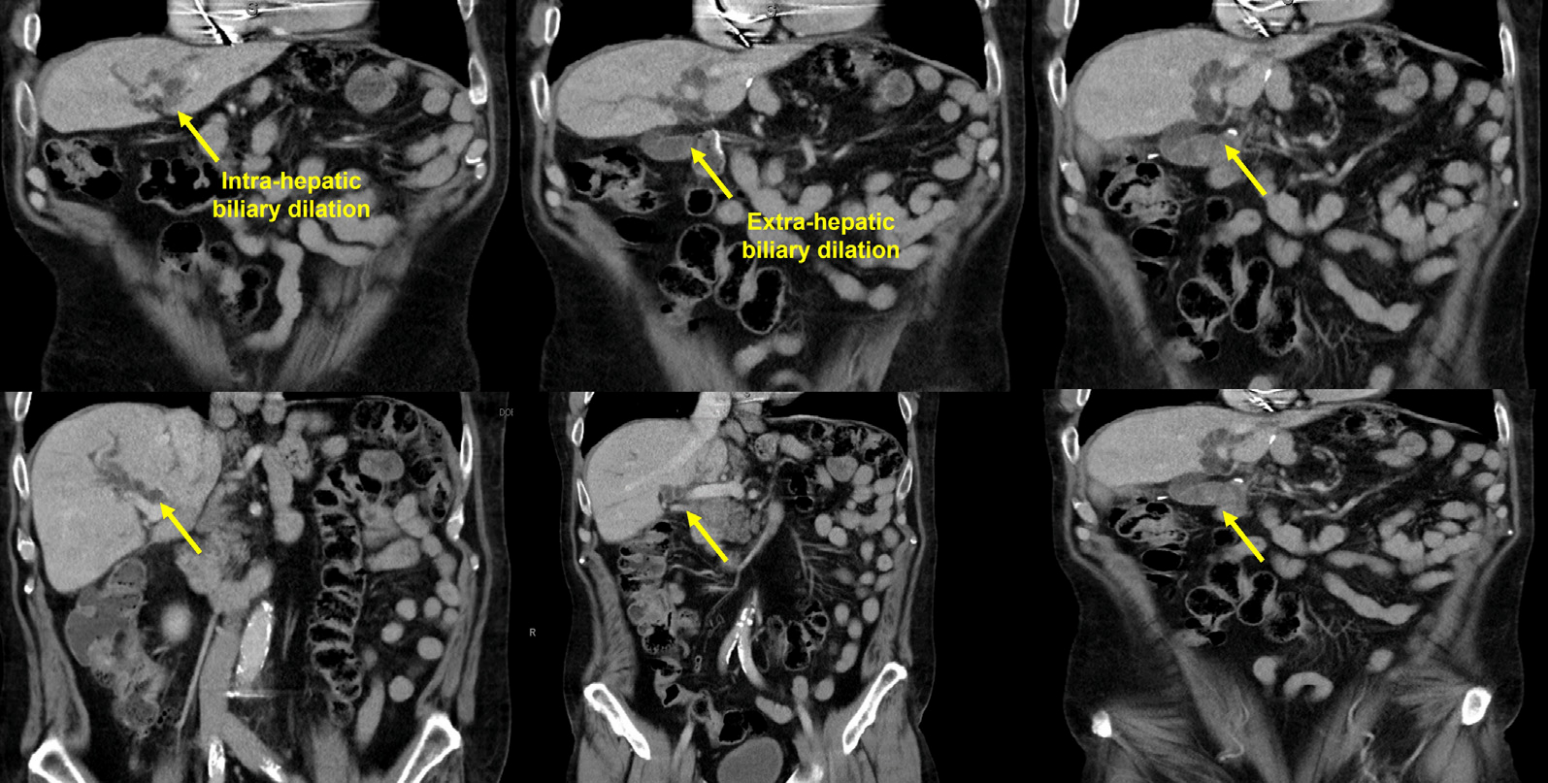
Coronal plane liver CT scan indicating dilation of the intrahepatic and extrahepatic bile ducts.

**Figure 3:**
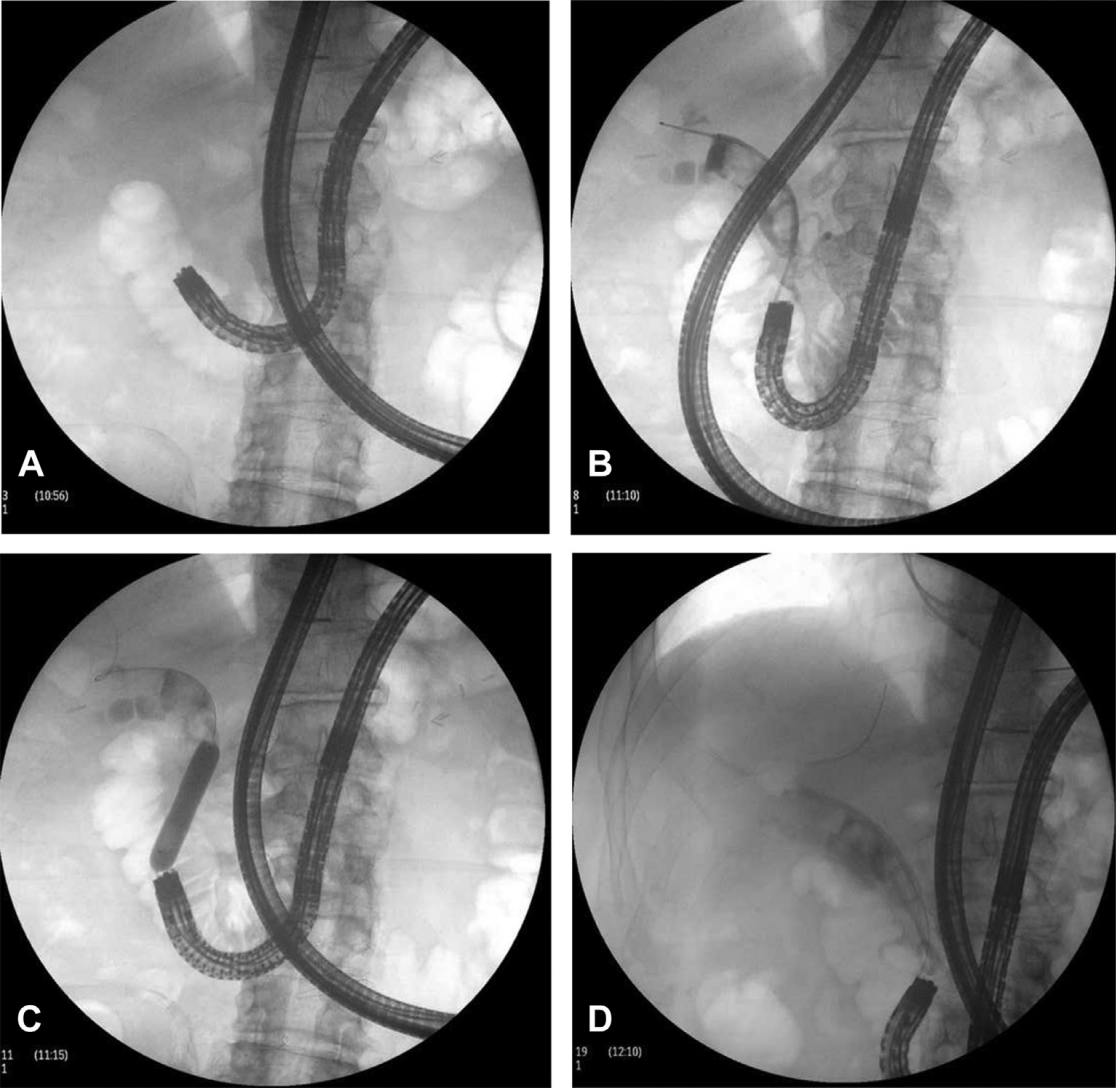
Fluoroscopic view of enteroscopy-assisted ERCP. **A**, Enteroscope advanced into the afferent limb at the site of the major papilla anastomosis. **B**, Diagnostic cholangiogram revealing multiple large biliary stones. **C**, Balloon dilation of the biliary orifice. **D**, Placement of multiple plastic biliary plastic stents.

A 67-year-old man with a history of total gastrectomy followed by Roux-en-Y esophagojejunostomy reconstruction in the setting of gastric adenocarcinoma presented with right-upper-quadrant pain and an abnormal liver function test (LFT) (aspartate aminotransferase 389, alanine aminotransferase 273, alkaline phosphatase 297, total bilirubin 8.70). A liver CT scan was consistent with dilation of the intrahepatic and extrahepatic bile ducts (Figs. 1 and 2; Video 1, available online at www.giejournal.org). The patient initially underwent enteroscopy-assisted ERCP, which revealed extensive choledocholithiasis (5 large stones) with a possible terminal bile duct stricture. Despite the multiple attempts to extract the stones with balloon sweeps after extending the sphincterotomy and performing sphincteroplasty, extraction was not possible, and the stone burden was not resolved (Fig. 3; Video 1). Therefore, the decision was made to place multiple biliary plastic stents and admit the patient for overnight observation.

In patients with surgically altered upper gastrointestinal anatomy, standard ERCP becomes technically challenging and therapeutic interventions are often limited by the length of these devices. Often, more invasive endoscopic, percutaneous, or even surgical approaches^1^, ^2^, ^3^, ^4^, ^5^ are needed to achieve the planned intervention. The decision is guided by available expertise, patient preference, and anticipated interventions.^6^ Because of the size and burden of the stones and the limited therapeutic devices that can be used through a colonoscope to treat large stones, 3 options were discussed with the patient: (1) repeat enteroscopy-assisted ERCP with plans to reattempt stone extraction with traditional techniques and replacement of multiple biliary plastic stents. Multiple repeated procedures were expected with time to ductal clearance uncertain. (2) Percutaneous transhepatic biliary drainage with subsequent cholangioscopy and lithotripsy through the percutaneous transhepatic cholangiography tract to achieve ductal clearance. This option was limited by the need for external drainage, which was opposed by the patient. (3) Creation of an enteroenterostomy using a lumen-apposing metal stent (LAMS) to facilitate cholangioscopy and lithotripsy. Despite the technically challenging nature of this procedure, it was deemed to be the better option for the patient given that it would allow advanced interventions, such as cholangioscopy with electrohydraulic lithotripsy (EHL), without the need for external drainage.

## Index Procedure

**Figure 4:**
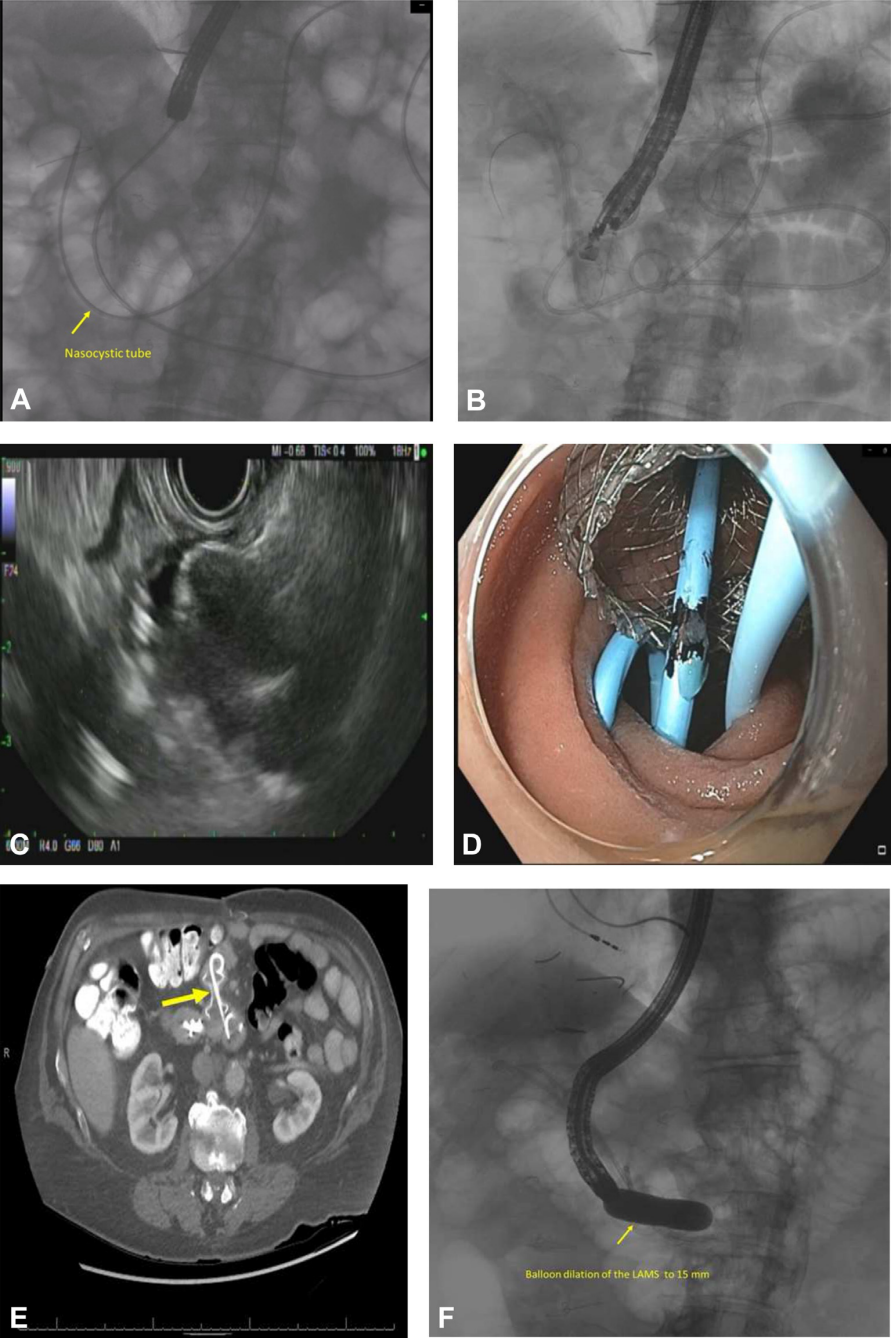
EUS-guided jejuno-jejunostomy with electrocautery-assisted 15- × 10-mm LAMS in a patient with total gastrectomy with Roux-en-Y esophagojejunostomy to facilitate cholangioscopy with electrohydraulic lithotripsy. **A**, A fluoroscopic image showing the orojejunal tube positioned to opacify the afferent limb. **B**, Fluoroscopic image indicating the EUS scope positioned in the roux limb. **C**, Under EUS guidance, the distal flange of the LAMS is deployed followed by the proximal flange using a free hand method. **D**, Endoscopic view of the deployed LAMS with a plastic stent traversing it. **E**, A coronal CT image confirming successful creation of the jejuno-jejunostomy. **F**, Balloon dilation of the LAMS to 15 mm 4 weeks after deployment. *LAMS*, Lumen-apposing metal stent.

**Figure 5:**
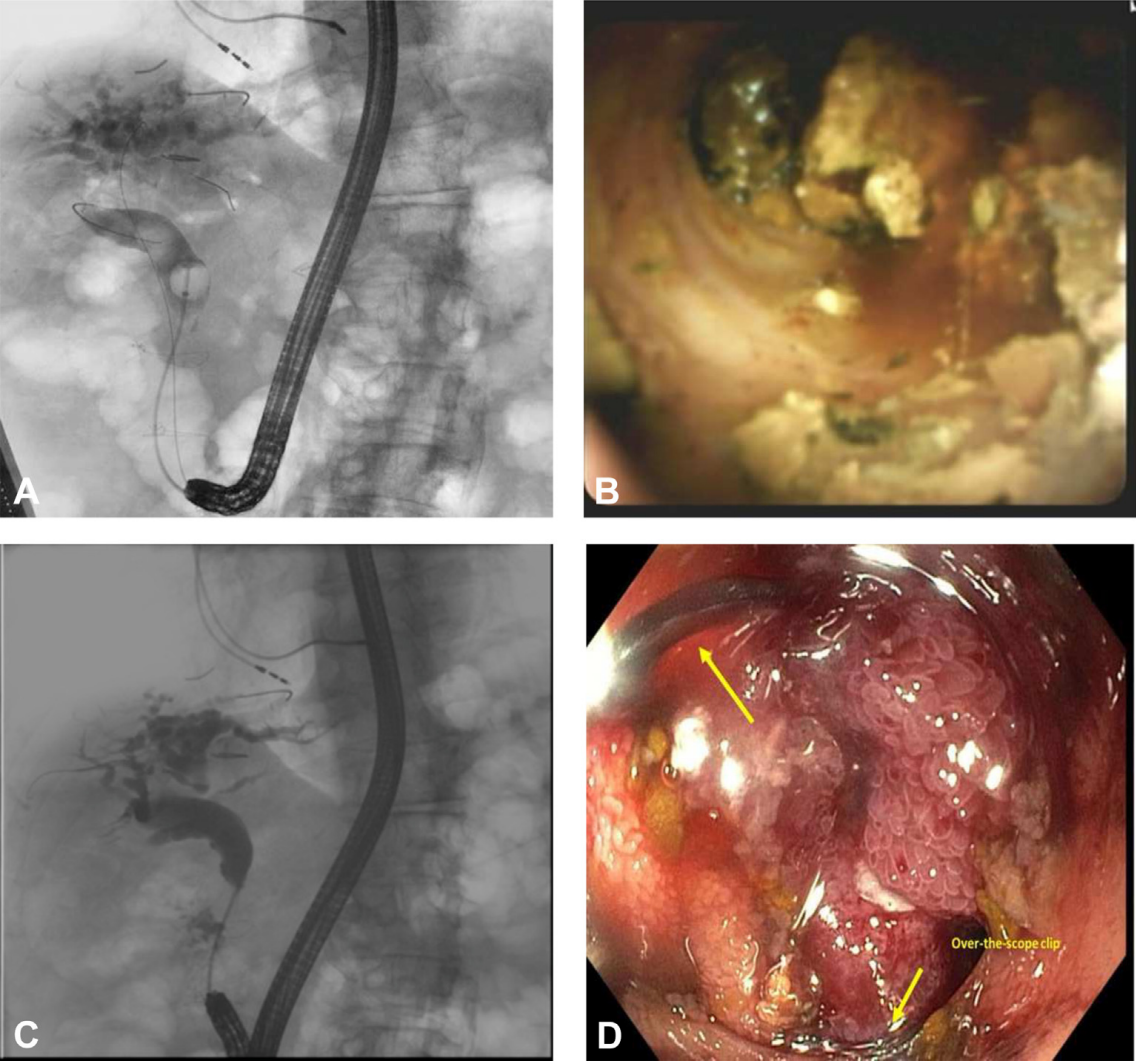
ERCP with cholangioscopy and electrohydraulic lithotripsy through the EUS-guided jejuno-jejunostomy. **A**, Fluoroscopic view of a diagnostic cholangiogram indicating the multiple large stones. **B**, Endoscopic image during cholangioscopy. **C**, Cholangiogram post–electrohydraulic lithotripsy indicating the absence of stones. **D**, Endoscopic closure of the jejuno-jejunostomy with argon plasma coagulation and an over-the-scope clip.

Initially, an orojejunal tube was placed in the afferent limb and used to inject contrast to opacify this limb. Using EUS, which was positioned in the roux limb and oriented toward the afferent limb, we deployed a 15- × 10-mm electrocautery-assisted LAMS creating a de novo jejuno-enterostomy, using a free-hand method. A plastic double-pigtail stent was placed through the LAMS to prevent stent-related erosion in the jejunum (Fig. 4; Video 1). The patient was admitted for an overnight observation, which was subsequently uncomplicated. Given that the ERCP was nonurgent and as multiple plastic stents were maintaining adequate biliary drainage, repeat ERCP was scheduled for 4 weeks later. During the follow-up ERCP, the previously placed plastic pigtail stent was removed, and the LAMS was dilated to 15 mm because of incomplete expansion. Ultimately, the fistula was traversed under both endoscopic and fluoroscopic guidance with a therapeutic gastroscope. The entry point into the afferent limb was just distal to the papilla, which was successfully canulated by gentle angulation of the gastroscope under fluoroscopic guidance followed by ERCP with cholangioscopy and EHL (Fig. 5). The large stones were successfully fragmented and 3 new plastic stents (one 7F and two 10F) were placed to ensure adequate biliary drainage. The patient’s procedure was uncomplicated, but he returned 4 weeks later for a repeat ERCP based on the complaint of reflux. During this procedure, his biliary stents were removed, and a high-quality cholangiogram confirmed complete ductal clearance with good contrast drainage. Because of the patient’s symptom of reflux, which was believed to be from bile reflux, we elected to actively close the enteroenterostomy. The previously deployed LAMS was removed, the edges of the fistulous tract were devitalized with argon plasma coagulation, and an over-the-scope clip was placed (Fig. 5; Video 1), leading to successful closure. The resolution of the fistula post-procedurally was not confirmed.

## Different Approaches That Are Commonly Used to Distend and Localize the Afferent Loop

### Use of orojejunal tube

This was the approach used in our case. It involves advancing a long orojejunal tube into the afferent loop with an enteroscope through which a mixture of saline solution, contrast, and methylene blue is injected to opacify the target/afferent loop.

### Enteroscopy assisted

In this approach, the afferent limb is reached with an enteroscope, and a solution containing a mixture of saline solution, contrast, and methylene blue is injected to opacify the target/afferent loop. This approach is often technically challenging and time consuming because it requires maneuvering the enteroscope through the angulations.

### Direct EUS puncture

In this approach, the afferent limb is identified under EUS guidance by following the common bile duct insertion site into the small-bowel loop and directly punctured with a 19-gauge needle followed by contrast injection. Direct visualization of the contrast in the afferent loop confirms the access to the target limb.

## Conclusion

During a clinical follow-up after 1 year, the patient had remained asymptomatic and had persistently normal LFTs. The creation of an EUS-guided jejuno-enterostomy with a LAMS to facilitate ERCP and cholangioscopy in altered anatomy appears to be a feasible option.

## Disclosure

*Dr Zuchelli is a consultant for Boston Scientific. All other authors disclosed no financial relationships.*

## Supplementary data

Video 1 EUS-guided jejuno-jejunostomy in a 67-year-old male patient with total gastrectomy with Roux-en-Y esophagojejunostomy to facilitate cholangioscopy with electrohydraulic lithotripsy.
